# Adverse childhood experiences and 10-year depressive-symptoms trajectories among middle-aged and older adults in China: a population-based cohort study

**DOI:** 10.3389/fpubh.2024.1455750

**Published:** 2024-12-09

**Authors:** Jin Xu, Guangxue Han, Xiulian Xu

**Affiliations:** ^1^School of Nursing and Rehabilitation, Shandong University, Jinan, Shandong, China; ^2^School of Nursing, Shandong University of Traditional Chinese Medicine, Jinan, Shandong, China; ^3^Department of Surgical Clinic, Qilu Hospital of Shandong University, Jinan, Shandong, China; ^4^Nursing Theory and Practice Innovation Research Center, Shandong University, Jinan, Shandong, China

**Keywords:** ACEs, CHARLS, life course epidemiology, depressive symptoms, trajectory

## Abstract

**Background:**

Adverse childhood experiences (ACEs) influence depressive symptoms. Depressive symptoms were heterogeneous from the perspective of life course.

**Objective:**

To explore the effects of ACEs on the trajectory of depressive symptoms in China.

**Participants:**

The data is from the 5 waves of the China Health and Retirement Longitudinal Study (CHARLS) and the 2014 Life Course Survey of it. A total of 17,106 individuals were included, without the people younger than 45 years.

**Methods:**

We dealt with the missing values using multiple interpolation. The CESD-10 and a 12-item questionnaire was used to assess the depressive symptom and ACEs, respectively. We used group-based trajectory modelling (GBTM) to identify the 10-year depressive-symptoms. Logistic regression models were used to explore associations between the trajectory and the ACEs.

**Results:**

Five depressive-symptom trajectories were identified based on the GBTM analysis (BIC = 540533.61; AIC = 540347.68; *n* = 17,106). Compared to the participants without depressive symptoms, the older adults who have more adverse childhood experiences have more odds of being in the other four groups, and the more ACEs the older adults experienced, the more likely it is.

**Conclusion:**

The 10-year depressive-symptoms trajectories among middle-aged and older adults in China were different from previous features. The significance of a life-course intervention plan to prevent childhood adversity and the related mental health damage in later life is demonstrated by the long-term influence of ACEs on depressive symptoms.

## 1 Introduction

With the gradual decline in fertility and the gradual increase in life expectancy, the proportion of older people in world is increasing. As an important part of the healthy aging strategy, mental health has been a hot research issue in the field of aging health (1). Since depression is one of the most prevalent mental illnesses and the second largest cause of disability globally (2), we need to pay special attention to depressive symptoms in the field of public health, which are the precursors of clinical depression. Depressive symptoms in older adults are associated with adverse consequences of multiple health outcomes (3), seriously affecting the social participation of the older adults, and impairing their social function, which ultimately increases the country’s health care burden (4). China is a large country with rapid development of aging, the depressive symptoms of Chinese older adults need urgent attention.

More and more ageing research was conducted from the perspective of life-course, which emphasize the importance of an accumulation of exposure of risk and the impact with age (5). Childhood is the critical period of development and growth in life. Adverse childhood experiences (ACEs) refer to various abuse experiences occurring during childhood or adolescence and influence the health (6). Being in a disadvantaged position in childhood will not only affect the development of the childhood (7), but also affect the health status in the middle and late adulthood (6, 8, 9).

Felitti’s team was the first to explore the association between adverse childhood experiences and depression risk (6). The effect of ACEs on depression in later life has been found in countries of all income levels, according to cumulative data (10, 11). Furthermore, a meta-analysis (12) revealed that compared to the people who did not experience ACEs, those who experienced more than there ACEs had a fourfold increased risk of depression. Research (13) has demonstrated that exposure to ACEs has a detrimental impact on people’ mental health and may increase their chance of developing depression. According to data from a recent study (14), early stressors can have a negative impact on mental health that lasts into old age. A research studying (15) the relationship between adverse childhood experiences and the sick age of bipolar disorder found that the age of illness significantly decreased with the occurrence of adverse childhood experiences. Another study (16) also indicates that the greater the number adverse childhood experiences were associated with an earlier age of mental illness. ACEs may worsen depressive symptoms in later life if the serious physiological and psychiatric illnesses were unsolved. In light with the previous study (16, 17), we included 12 ACEs from CHARLS, because the most widely used 10-item ACEs from the CDC–Kaiser Permanente ACE Study were generated based on a sample of mostly White and educated individuals, which may not adequately apply to the Chinese populations (18).

Behavioral trajectories refer to the processes that change behavior with age or time. These group distinctions have significant effects on how we comprehend the fundamental cause of depression. Distinct trajectory patterns could be a sign of underlying etiological variations. Additionally, people who exhibit specific long-term trajectory patterns may bear a disproportionate share of the harmful effects and contribute to the public health burden of depression. One study showed that depression trajectories vary across the population over time (19). Although existing studies in China have explored the effects of childhood adversity on depressive symptoms (20), but the effect on the depression trajectory was not explored. So it is worthwhile to examine the relationship between ACEs and long-term patterns of depressive symptoms. A group-based approach (21) can be used to identify individual depression trajectories for specific groups.

## 2 Methods

### 2.1 Study design and respondents

The China Health and Retirement Longitudinal Study (CHARLS) (22) was a nationally representative longitudinal survey among the residents aged 45 and older in China, which recruited participants from 150 counties in 28 provinces in China (23). A standardized questionnaire was used to collect data on participants’ sociodemographic characteristics and health-related information (23). All participants were followed up every 2 years and 4 subsequent follow-ups have been carried out.

We used the data of baseline and the four follow-ups, as well as the data from the 2014 Life Course Survey. A total of 17,708 participants were recruited at baseline in 2011. People younger than 45 years were excluded, and a total of 17,106 individuals were included in the study.

The Biomedical Ethics Review Committee of Peking University approved the CHARLS study (IRB00001052–11015), and all the data is publicly available.

### 2.2 Measurements

#### 2.2.1 Depressive symptom

Depressive symptoms in the 5-wave survey were assessed using the 10-item Center for Epidemiologic Studies Depression Scale (CESD-10) (24). We used this scale to assess how often participants experienced any of the 10 depressive symptoms in the past week (24). This is a 4-category scale, with each item assigned a score of 0–3 for a total score of 30, with higher total scores representing more severe depressive symptoms (23). It is important to note that questions 5 and 8 of this scale are scored backwards (23). According to a previous study (16, 41), those with a score of 12 or more are defined as having depressive symptoms.

#### 2.2.2 Adverse childhood experiences (ACEs)

Conventional ACEs (25) consisted of 7 domains, including physical abuse, emotional neglect, family substance abuse, family mental illness, domestic violence, incarceration of family members, parental separation, or divorce (26). An additional set of expanded ACEs (27) consisted of 2 domains, including unsafe neighborhood and bullying. Several new ACEs (21, 28) indicators were included recently, consisting of parental death, sibling death and parental disability, 3 domains. The details of the 12 definitions of ACEs and the questionnaire items are shown in Supplementary Table S1. Participants’ responses to each ACE item were dichotomized (assigned values of 0 / 1) and summed to generate a cumulative ACE score for each participant, ranging from 0 to 12. We further categorized participants into 5 groups based on the cumulative ACE scores (17): 0, 1, 2, 3, and 4 or higher.

#### 2.2.3 Individual-level covariates

Control variables included age, sex, marital status, residence, education, the self-reported health, multiple diseases coexist, smoking status and alcohol drinking status.

### 2.3 Statistical analysis

Descriptive analysis and the trajectory modelling was conducted using Stata17.0 and Mplus 8.3 software.

First, the missing values were processed by multiple interpolation. We used group-based trajectory modelling to identify distinct patterns of depressive symptom levels according to the depressive symptom scores among 5 waves. This research approach allowed us to estimate the probability of multiple trajectories between different groups of individuals, rather than modeling a single average for the study population (29). To determine the optimal number of trajectories of depressive symptoms, we fitted 6 sets of trajectories models, including 1 group to 6 groups. We selected the best-fit model based on the following criteria: (1) the average posterior probability for each group of trajectories was ≥0.70 (30); (2) the sample size of each trajectory set should exceed 5.0% of the total sample size; and (3) the case with the lowest absolute value of the Bayesian Information Criterion (BIC) was selected (31). According to the above criteria, a model of 5 trajectory groups was determined.

Descriptive features of subjects in the different depressive symptom trajectories were compared through the analysis of *χ*^2^ tests for categorical variables. Logistic regression models were used to explore associations between the trajectory groups of depressive symptom and the adverse childhood experiences. We used the following two sets of model: Model 1 was the raw model. Model 2 was adjusted by categorical variables.

## 3 Results

There should be 5 groups for depressive symptoms using the GBTM analysis (BIC = 540533.61; AIC = 540347.68; *n* = 17,106). For the reason that, the population percentage of one among six groups is 4.04%, which is less than 5% and does not fulfill the standards. The absolute value of Bayesian information criterion and Akaike’s information criterion is the most mini in the 5-group model. And all 5 classes have an average posterior probability higher than 0.70. More details are shown in Table 1.

**Table 1:** Fit of the different subgroup depressive-symptoms trajectory models.

Group	1	2	3	4	5	6
AIC	555104.53	544052.31	542493.85	540952.29	540347.68	539884.04
BIC	555104.52	544145.28	542617.80	541107.23	540533.61	540100.96
Class 1	100	77.23	30.79	6.00	57.34	12.21
Class 2		22.77	5.68	16.70	13.09	13.13
Class 3			63.53	59.73	6.23	5.83
Class 4				17.59	13.11	54.58
Class 5					10.23	10.21
Class 6						4.04
Class 1	1.00	0.95	0.80	0.86	0.90	0.73
Class 2		0.88	0.84	0.78	0.73	0.73
Class 3			0.90	0.91	0.85	0.69
Class 4				0.76	0.75	0.89
Class 5					0.72	0.71
Class 6						0.82

**Figure 1:**
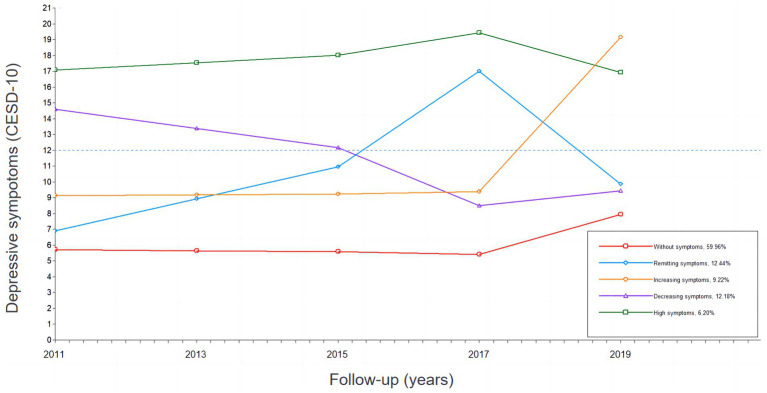
10-year depressive-symptoms trajectories among middle-aged and older adults in China.

Five depressive-symptom trajectories were identified, including “Without symptoms” (*n* = 10,257; 59.96%); “Decreasing symptoms” (*n* = 2,083; 12.18%); “High symptoms” (*n* = 1,061; 6.20%); “Remitting symptoms” (*n* = 2,128; 12.44%); “Increasing symptoms” (*n* = 396; 9.22%). The trajectories was shown in Figure 1.

The baseline characteristics of the 5 depressive symptoms trajectory groups are shown in Table 2. About half of the participants were men, 48.79% lived in rural areas, and 86.99% were married. Most (38.91%) have an education level of Primary school. A larger proportion (38.59%) suffer from two or more chronic diseases. A minority (5.91%) live alone. Only one-quarter of the older adults did not experience ACEs. According to the results of the *χ*^2^ tests, there were significant differences in age, sex, marital status, residence, education, the self-reported health, multiple diseases coexist, smoking status and alcohol drinking status (all *p* < 0.05).

**Table 2:** Characteristics of participants according to different depressive-symptoms trajectories.

Characteristics	Overall (*n* = 17,106)	Without symptoms	Decreasing symptoms	High symptoms	Remitting symptoms	Increasing symptoms	*p* value
*N* (%)	100	10,257 (59.96)	2,083 (12.18)	1,061 (6.20)	2,128 (12.44)	396 (9.22)	–
Age group							<0.001
45–59	9,650 (56.41)	6,011 (58.60)	1,027 (49.30)	594 (55.98)	1,154 (54.23)	864 (54.79)	
60–74	5,887 (34.41)	3,366 (32.82)	789 (37.88)	420 (39.59)	748 (35.15)	564 (35.76)	
75–89	1,364 (7.97)	763 (7.44)	235 (11.28)	43 (4.05)	199 (9.35)	124 (7.86)	
≥89	205 (1.20)	117 (1.14)	32 (1.54)	4 (0.38)	27 (1.27)	25 (1.59)	
Gender							<0.001
Female	8,760 (51.21)	4,670 (45.53)	1,243 (59.67)	768 (72.38)	1,189 (55.87)	890 (56.44)	
Male	8,346 (48.79)	5,587 (54.47)	840 (40.33)	293 (27.62)	939 (44.13)	687 (43.56)	
Residence							<0.001
Urban	6,942 (40.58)	4,795 (46.75)	637 (30.58)	248 (23.37)	757 (35.57)	505 (32.02)	
Rural	10,164 (59.42)	5,462 (53.25)	1,446 (69.42)	813 (76.63)	1,371 (64.43)	1,072 (67.98)	
Marital status							<0.001
Married	14,881 (86.99)	9,154 (89.25)	1,675 (80.41)	877 (82.66)	1,833 (86.14)	1,342 (85.10)	
Not married	2,225 (13.01)	1,103 (10.75)	408 (19.59)	184 (17.34)	295 (13.86)	235 (14.90)	
Educational level							<0.001
Illiterate	4,736 (27.69)	2,331 (22.73)	781 (37.49)	452 (42.60)	658 (30.92)	514 (32.59)	
Primary school	6,656 (38.91)	3,852 (37.55)	838 (40.23)	426 (40.15)	875 (41.12)	665 (42.17)	
Middle school	3,526 (20.61)	2,426 (23.65)	316 (15.17)	137 (12.91)	365 (17.15)	282 (17.88)	
High school or above	2,188 (12.79)	1,648 (16.07)	148 (7.11)	46 (4.34)	230 (10.81)	116 (7.36)	
Multiple diseases coexist (%)							<0.001
No	10,504 (61.41)	6,930 (67.56)	1,012 (48.58)	413 (38.93)	1,257 (59.07)	892 (56.56)	
Yes	6,602 (38.59)	3,327 (32.44)	1,071 (51.42)	648 (61.07)	871 (40.93)	685 (43.44)	
Living alone							
No	16,095 (94.09)	9,703 (94.60)	1,929 (92.61)	990 (93.31)	1,991 (93.56)	1,482 (93.98)	
Yes	1,011 (5.91)	554 (5.40)	154 (7.39)	71 (6.69)	137 (6.44)	95 (6.02)	
Smoking status							<0.001
Never smoker	10,615 (62.05)	6,096 (59.43)	1,364 (65.48)	771 (72.67)	1,365 (64.14)	1,019 (64.62)	
Former smoker	1,473 (8.61)	951 (9.27)	165 (7.92)	71 (6.69)	162 (7.61)	124 (7.86)	
Current smoker	5,018 (29.33)	3,210 (31.30)	554 (26.60)	219 (20.64)	601 (28.24)	434 (27.52)	
Drinking status							<0.001
Never drinker	9,977 (58.32)	5,675 (55.33)	1,329 (63.80)	729 (68.71)	1,293 (60.76)	951 (60.30)	
Former drinker	1,447 (8.46)	796 (7.76)	213 (10.23)	95 (8.95)	203 (9.54)	140 (8.88)	
Current drinker	5,682 (33.22)	3,786 (36.91)	541 (25.97)	237 (22.34)	632 (29.70)	486 (30.82)	
SRH							
Excellent/very good	1,274 (7.45)	986 (9.61)	63 (3.02)	13 (1.23)	130 (6.11)	82 (5.20)	
Good	2,767 (16.18)	2,066 (20.14)	202 (9.70)	53 (5.00)	264 (12.41)	182 (11.54)	
Fair	7,958 (46.52)	5,109 (49.81)	756 (36.29)	324 (30.54)	1,030 (48.40)	739 (46.86)	
Poor/very poor	5,107 (29.86)	2,096 (20.43)	1,062 (50.98)	671 (63.24)	704 (33.08)	574 (36.40)	
ACEs score							<0.001
0	4,333 (25.33)	2,854 (27.82)	432 (20.74)	161 (15.17)	527 (24.77)	359 (22.76)	
1	5,372 (31.40)	3,422 (33.36)	610 (29.28)	260 (24.51)	617 (28.99)	463 (29.36)	
2	3,816 (22.31)	2,216 (21.60)	487 (23.38)	254 (23.94)	473 (22.23)	386 (24.48)	
3	2,126 (12.43)	1,121 (10.93)	308 (14.79)	197 (18.57)	289 (13.58)	211 (13.38)	
≥4	1,459 (8.53)	644 (6.28)	246 (11.81)	189 (17.81)	222 (10.43)	158 (10.02)	

The association between ACEs and the depressive-symptoms trajectory is shown in Table 3, which is adjusted. And the unadjusted version is included in the Supplementary Table S2. Compared to the participants without depressive symptoms, the older adults who have more adverse childhood experiences have more odds of being in the other four groups, and the more ACEs the older adults experienced, the more likely it is. However, there is no difference between the participants in “Without symptoms” and “Remitting symptoms,” or between “Without symptoms” and “Increasing symptoms,” when the participants have only one ACE. The association between 12 domains of ACEs and depressive-symptoms trajectory is shown in Table 4, which is adjusted, too. The unadjusted version is in the Supplementary Table S3.

**Table 3:** Association between ACEs and depressive-symptoms trajectory.

ACEs	OR^a^ =*e^β^* (95% CI)^b^			
	Decreasing symptoms	High symptoms	Remitting symptoms	Increasing symptoms
0	1.00	1.00	1.00	1.00
1	1.21 (0.06, 0.33)	1.39 (0.12, 0.54)	0.99 (−0.15, 0.10)	1.09 (−0.06, 0.23)
2	1.45 (0.22, 0.51)	1.96 (0.46, 0.89)	1.16 (0.01, 0.28)	1.37 (0.16, 0.47)
3	1.75 (0.39, 0.73)	2.91 (0.84, 1.30)	1.38 (0.16, 0.48)	1.44 (0.18, 0.55)
≥4	2.46 (0.71, 1.09)	4.94 (1.36, 1.84)	1.87 (0.44, 0.81)	1.90 (0.43, 0.85)

a Adjusted for age, sex, marital status, residence, education, the self-reported health, multiple diseases coexist, smoking status and alcohol drinking status.

b Reported 95% CI correspond to β; significance threshold = 0.

**Table 4:** Association between 12 domains of ACEs and depressive-symptoms trajectory.

Domains		OR^a^ =*e^β^* (95% CI)^b^			
		Decreasing symptoms	High symptoms	Remitting symptoms	Increasing symptoms
Physical abuse	No	1.00	1.00	1.00	1.00
	Yes	1.30 (0.15, 0.37)	1.59 (0.32, 0.61)	1.10 (−0.02, 0.20)	1.18 (0.04, 0.29)
Emotional neglect	No	1.00	1.00	1.00	1.00
	Yes	1.08 (−0.03, 0.18)	1.23 (0.07, 0.35)	1.08 (−0.03, 0.17)	1.07 (−0.05, 0.18)
Household substance abuse	No	1.00	1.00	1.00	1.00
	Yes	1.05 (−0.15, 0.24)	1.27 (−0.01, 0.48)	0.86 (−0.35, 0.05)	0.97 (−0.25, 0.19)
Household mental illness	No	1.00	1.00	1.00	1.00
	Yes	1.83 (0.46, 0.74)	3.40 (1.06, 1.38)	1.69 (0.39, 0.67)	1.55 (0.28, 0.60)
Domestic violence	No	1.00	1.00	1.00	1.00
	Yes	1.44 (0.19, 0.54)	1.85 (0.39, 0.83)	1.05 (−0.14, 0.24)	1.37 (0.12, 0.52)
Incarcerated household member	No	1.00	1.00	1.00	1.00
	Yes	1.07 (−1.04, 1.16)	0.60 (−2.56, 1.55)	3.00 (0.38, 1.81)	2.46 (0.03, 1.77)
Parental separation divorce	No	1.00	1.00	1.00	1.00
	Yes	1.70 (0.07, 0.99)	1.19 (−0.50, 0.85)	0.97 (−0.59, 0.54)	1.62 (−0.03, 1.00)
Unsafe neighborhood	No	1.00	1.00	1.00	1.00
	Yes	1.31 (0.10, 0.44)	2.01 (−0.50, 0.90)	1.17 (−0.01, 0.33)	1.50 (0.22, 0.58)
Bullying	No	1.00	1.00	1.00	1.00
	Yes	1.50 (0.27, 0.54)	2.18 (0.61, 0.95)	1.31 (0.14, 0.41)	1.34 (0.12, 0.44)
Parental death	No	1.00	1.00	1.00	1.00
	Yes	1.44 (0.25, 0.48)	1.97 (0.53, 0.82)	1.24 (0.10, 0.34)	1.43 (0.15, 0.41)
Sibling death	No	1.00	1.00	1.00	1.00
	Yes	0.98 (−0.14, 0.11)	1.15 (−0.03, 0.31)	0.95 (−0.18, 0.07)	0.92 (−0.23, 0.06)
Parental disability	No	1.00	1.00	1.00	1.00
	Yes	1.07 (−0.15, 0.28)	1.08 (−0.21, 0.37)	0.95 (−0.27, 0.16)	0.91 (−0.35, 0.15)

a Adjusted for age, sex, marital status, residence, education, the self-reported health, multiple diseases coexist, smoking status and alcohol drinking status.

b Reported 95% CI correspond to β; significance threshold = 0.

## 4 Discussion

This study was a population-based investigation of the impact of ACEs on long-term depressive-symptoms trajectories in a nationally representative sample of middle-aged and older Chinese adults. Five depressive-symptoms trajectories were identified. The trajectory characteristics of this study has some similarities with the previous study (19). Such as, there were both 5 groups. The trend for the first 8 years of the 10-year trajectories is generally consistent with the 8-year trajectories of the previous study (19), relatively flat. However, the trajectory of the later 2 years did not continue the earlier trends. Surprisingly, the 10-year trajectories of depressive symptoms, which based on the CHARLS, were largely consistent with that from Netherlands, which based on the Rotterdam Study (32). To some extent, this may suggest that there does not appear to be a difference in the long-term trajectory of depressive symptoms for the middle-aged and older adults across countries and races.

In this study, most (59.96%) had a cesd-10 score consistently below 12 and were defined as “Without symptoms.” Few (6.20%) had a cesd-10 score consistently above 12 and were defined as “High symptoms.” Those with a slowing overall trend in depressive symptoms were defined as “Decreasing symptoms.” And those with an increase trend were defined as “Increasing symptoms.” Interestingly, there was a group with a sharp decline below 12 after a sustained rise in depressive symptoms, which was defined as “Remitting symptoms.”

A large proportion of older adults people have experienced ACEs in China, and up to 3/4 of them experienced more than one, which is consistent with previous research (16). We found that participants who experienced more ACEs were at greater risk for classification into “High symptoms” and “Increasing symptoms,” suggesting a cumulative effect of the amount of ACEs on the effects of depression. So it is necessary to focus on the impact of ACEs on the health of middle-aged and older adults people in China.

The participants whose female/male guardian had alcoholism/drug or had abnormality of mind when they were young were more likely to be classified into “High symptoms”, as shown in Table 4. In other words, household mental illness and substance abuse during childhood can make them vulnerable to depression in later life, which is in line with the previous studies (33, 34). One plausible explanation is that childhood familial circumstances, including genetic makeup and living conditions, have enduring impacts on children’s health (35).

In addition, we observed that an increased incidence of adult depression has been linked to childhood physical abuse, violence (domestic violence or hazardous neighborhoods), and peer bullying, which is in line with the literature (35, 36). Additionally, kids who are physically abused could be more likely bullied by others, which could alter stress reactions or cause persistent elevations in inflammatory processes, increasing their chance of developing depression later in life (17, 37).

In line with earlier research (6), our results showed that having experienced an ACE was linked to a higher chance of developing depressive symptoms later in life. Those who had one or more ACEs were more likely to experience depressive symptoms later in life than those who were not exposed. There are several possible mechanisms to explain such association. ACE-induced chronic toxic stress may cause hypothalamic–pituitary–adrenal (HPA) axis dysfunction, which has been linked to higher cortisol and glucocorticoid resistance levels (38). According to Heim (39), these metabolic alterations may eventually make a person more susceptible to depression symptoms. Besides, ACEs may cause dysregulation of several systems and an increase in allostatic stress, which may contribute to psychopathology, including depression (40). According to these results, a life-course public health approach may be used to lower the possible risks of late-life depression.

The present study has two advantages. First, we interpolated the missing values with multiple imputation to maximize the data completeness. Second, we investigated the influence of ACEs on the 10-year depression trajectory of the Chinese middle and later older adults from the perspective of life course, using a nationwide representative sample, which broke through the limitations of previous studies, found new trajectories that differed from previous features and provided important insights about the influence of ACEs across the life course. This study has several limitations. First, the data were self reported. Therefore, recall and measurement biases were unavoidable. Second, the ACEs frequency and intensity were not recorded. Future research is needed to keep track of the trajectory of depression in order to discover new trajectories.

Our study’s conclusions have significant ramifications for clinical practice. The significance of a life-course intervention plan to prevent childhood adversity and the related mental health damage in later life is demonstrated by the long-term influence of ACEs on depressive symptoms. The study indicates that preventive measures to reduce the incidence of ACEs are crucial for lowering the risk of depression in later life. These preventive measures may include raising public awareness about ACEs, providing early intervention and support, and implementing public health strategies to reduce the occurrence of ACEs and mitigate their impact on individual mental health.

## Data Availability

The datasets presented in this study can be found in online repositories. The names of the repository/repositories and accession number(s) can be found at: http://charls.pku.edu.cn/index/en.html.
