# Correction: Adverse childhood experiences and 10-year depressive-symptoms trajectories among middle-aged and older adults in China: a population-based cohort study

**DOI:** 10.3389/fpubh.2025.1656002

**Published:** 2025-08-05

**Authors:** Jin Xu, Guangxue Han, Xiulian Xu

**Affiliations:** ^1^School of Nursing and Rehabilitation, Shandong University, Jinan, Shandong, China; ^2^School of Nursing, Shandong University of Traditional Chinese Medicine, Jinan, Shandong, China; ^3^Department of Surgical Clinic, Qilu Hospital of Shandong University, Jinan, Shandong, China; ^4^Nursing Theory and Practice Innovation Research Center, Shandong University, Jinan, Shandong, China

**Keywords:** ACEs, CHARLS, life course epidemiology, depressive symptoms, trajectory

In the published article, there was an error in Table 3 as published. The label b of “(95% CI)” was omitted. The corrected Table 3 and its caption appear below.

**Table 3:** Association between ACEs and depressive-symptoms trajectory.

**ACEs**	**OR^a^** = ***e***^**β**^ **(95% CI)^b^**			
	**Decreasing symptoms**	**High symptoms**	**Remitting symptoms**	**Increasing symptoms**
0	1.00	1.00	1.00	1.00
1	1.21 (0.06, 0.33)	1.39 (0.12, 0.54)	0.99 (−0.15, 0.10)	1.09 (−0.06, 0.23)
2	1.45 (0.22, 0.51)	1.96 (0.46, 0.89)	1.16 (0.01, 0.28)	1.37 (0.16, 0.47)
3	1.75 (0.39, 0.73)	2.91 (0.84, 1.30)	1.38 (0.16, 0.48)	1.44 (0.18, 0.55)
≥4	2.46 (0.71, 1.09)	4.94 (1.36, 1.84)	1.87 (0.44, 0.81)	1.90 (0.43, 0.85)

a Adjusted for age, sex, marital status, residence, education, the self-reported health, multiple diseases coexist, smoking status and alcohol drinking status.

b Reported 95% CI correspond to β; significance threshold = 0.

In the published article, there was an error in Table 4 as published. The label b of “(95% CI)” was omitted. The corrected Table 4 and its caption appear below.

**Table 4:** Association between 12 domains of ACEs and depressive-symptoms trajectory.

**Domains**		**OR^a^** = ***e***^**β**^ **(95% CI)^b^**			
		**Decreasing symptoms**	**High symptoms**	**Remitting symptoms**	**Increasing symptoms**
Physical abuse	No	1.00	1.00	1.00	1.00
	Yes	1.30 (0.15, 0.37)	1.59 (0.32, 0.61)	1.10 (−0.02, 0.20)	1.18 (0.04, 0.29)
Emotional neglect	No	1.00	1.00	1.00	1.00
	Yes	1.08 (−0.03, 0.18)	1.23 (0.07, 0.35)	1.08 (−0.03, 0.17)	1.07 (−0.05, 0.18)
Household substance abuse	No	1.00	1.00	1.00	1.00
	Yes	1.05 (−0.15, 0.24)	1.27 (−0.01, 0.48)	0.86 (−0.35, 0.05)	0.97 (−0.25, 0.19)
Household mental illness	No	1.00	1.00	1.00	1.00
	Yes	1.83 (0.46, 0.74)	3.40 (1.06, 1.38)	1.69 (0.39, 0.67)	1.55 (0.28, 0.60)
Domestic violence	No	1.00	1.00	1.00	1.00
	Yes	1.44 (0.19, 0.54)	1.85 (0.39, 0.83)	1.05 (−0.14, 0.24)	1.37 (0.12, 0.52)
Incarcerated household member	No	1.00	1.00	1.00	1.00
	Yes	1.07 (−1.04, 1.16)	0.60 (−2.56, 1.55)	3.00 (0.38, 1.81)	2.46 (0.03, 1.77)
Parental separation divorce	No	1.00	1.00	1.00	1.00
	Yes	1.70 (0.07, 0.99)	1.19 (−0.50, 0.85)	0.97 (−0.59, 0.54)	1.62 (−0.03, 1.00)
Unsafe neighborhood	No	1.00	1.00	1.00	1.00
	Yes	1.31 (0.10, 0.44)	2.01 (−0.50, 0.90)	1.17 (−0.01, 0.33)	1.50 (0.22, 0.58)
Bullying	No	1.00	1.00	1.00	1.00
	Yes	1.50 (0.27, 0.54)	2.18 (0.61, 0.95)	1.31 (0.14, 0.41)	1.34 (0.12, 0.44)
Parental death	No	1.00	1.00	1.00	1.00
	Yes	1.44 (0.25, 0.48)	1.97 (0.53, 0.82)	1.24 (0.10, 0.34)	1.43 (0.15, 0.41)
Sibling death	No	1.00	1.00	1.00	1.00
	Yes	0.98 (−0.14, 0.11)	1.15 (−0.03, 0.31)	0.95 (−0.18, 0.07)	0.92 (−0.23, 0.06)
Parental disability	No	1.00	1.00	1.00	1.00
	Yes	1.07 (−0.15, 0.28)	1.08 (−0.21, 0.37)	0.95 (−0.27, 0.16)	0.91 (−0.35, 0.15)

a Adjusted for age, sex, marital status, residence, education, the self-reported health, multiple diseases coexist, smoking status and alcohol drinking status.

b Reported 95% CI correspond to β; significance threshold = 0.

In the published article, there was an error in Supplementary Table S2. The label of “(95% CI)” was omitted. The corrected Supplementary Table S2 and its caption appear below.

**Table S2:** Association between ACEs and depressive-symptoms trajectory.

	**OR** = **e**^**β**^ **(95% CI)^a^**			
**ACEs**	**Decreasing symptoms**	**High symptoms**	**Remitting symptoms**	**Increasing symptoms**
0	1.00	1.00	1.00	1.00
1	1.18 (0.03,0.30)	1.35 (0.10,0.50)	0.98 (−0.15,0.10)	1.08 (−0.07,0.22)
2	1.45 (0.23,0.51)	2.03 (0.50,0.91)	1.16 (0.01,0.28)	1.38 (0.17,0.48)
3	1.82 (0.43,0.76)	3.12 (0.92,1.36)	1.40 (0.17,0.49)	1.50 (0.22,0.59)
≥4	2.52 (0.75,1.10)	5.20 (1.42,1.88)	1.87 (0.45,0.80)	1.95 (0.46,0.87)

a Reported 95% CI correspond to β; significance threshold = 0.

In the published article, there was an error in Supplementary Table S3. The label of “(95% CI)” was omitted. The corrected Supplementary Table S3 and its caption appear below.

**Table S3:** Association between 12 domains of ACEs and depressive-symptoms trajectory.

**Domains**		**OR** = **e**^**β**^ **(95% CI)^a^**			
		**Decreasing symptoms**	**High symptoms**	**Remitting symptoms**	**Increasing symptoms**
Physical abuse	No	1.00	1.00	1.00	1.00
	Yes	1.01 (−0.10,0.13)	0.98 (−0.17,0.14)	0.93 (−0.18,0.05)	0.97 (−0.16,0.10)
Emotional neglect	No	1.00	1.00	1.00	1.00
	Yes	0.96 (−0.15,0.07)	1.00 (−0.15,0.14)	1.04 (−0.07,0.14)	0.99 (−0135,0.11)
Household substance abuse	No	1.00	1.00	1.00	1.00
	Yes	0.80 (−0.42,-0.02)	0.75 (−0.55,-0.02)	0.75 (−0.50,-0.08)	0.79 (−0.46,-0.01)
Household mental illness	No	1.00	1.00	1.00	1.00
	Yes	1.86 (0.47,0.77)	3.42 (1.06,1.40)	1.81 (0.45,0.75)	1.56 (0.27,0.62)
Domestic violence	No	1.00	1.00	1.00	1.00
	Yes	1.18 (−0.02,0.36)	1.27 (0.00,0.47)	0.94 (−0.27,0.14)	1.21 (−0.02,0.41)
Incarcerated household member	No	1.00	1.00	1.00	1.00
	Yes	0.65 (−1.51,0.65)	0.27 (−3.33,0.72)	2.39 (0.16,1.58)	1.63 (0.38,1.35)
Parental separation divorce	No	1.00	1.00	1.00	1.00
	Yes	1.68 (0.07,0.97)	1.08 (−0.58,0.74)	0.91 (−0.66,0.47)	1.52 (−0.10,0.94)
Unsafe neighborhood	No	1.00	1.00	1.00	1.00
	Yes	1.15 (−0.04,0.32)	1.46 (0.17,0.59)	1.09 (−0.09,0.27)	1.41 (0.15,0.53)
Bullying	No	1.00	1.00	1.00	1.00
	Yes	1.12 (−0.03,0.26)	1.31 (0.09,0.45)	1.13 (0.02,0.27)	1.08 (−0.09,0.24)
Parental death	No	1.00	1.00	1.00	1.00
	Yes	1.38 (0.20,0.44)	1.67 (0.36,0.67)	1.19 (0.05,0.30)	1.31 (0.13,0.41)
Sibling death	No	1.00	1.00	1.00	1.00
	Yes	0.90 (−0.24,0.02)	0.80 (−0.40,0.05)	0.88 (−0.26,0.01)	0.85 (−0.31,-0.01)
Parental disability	No	1.00	1.00	1.00	1.00
	Yes	0.90 (−0.32,0.11)	0.63 (−0.77,-0.15)	0.89 (−0.34,0.10)	0.77 (−0.52,0.01)

a Reported 95% CI correspond to β; significance threshold = 0.

The original version of this article has been updated.

