# Supplementary material for: Adverse childhood experiences and 10-year depressive-symptoms trajectories among middle-aged and older adults in China: a population-based cohort study
Source: Front Public Health. 2024 Dec 9;12:1455750. doi: 10.3389/fpubh.2024.1455750 (PMC11663716; doi:10.3389/fpubh.2024.1455750)
Supplement: Supplementary file 1 [file Data_Sheet_1.pdf]

Supplementary Material

Table S1 Questionnaire items of Each ACE indicator.

Types of ACEs	Domain	Questionnaire items
Conventional ACEs	Physical abuse	When you were growing up, did your female/male guardian ever hit you? (often[a], sometimes[a], rarely, or never)
	Emotional neglect	How much love and affection did your female guardian give you while you were growing up?(often[a], sometimes[a], rarely, or never)
		How much effort did your female guardian put into watching over you? (a lot, some, a little[a], or not at all[a])
	Household substance abuse	During the years you were growing up, did your female/male guardian ever have alcoholism or drug?(yes[a] or no)
	Household mental illness	Did your female/male guardian have abnormality of mind when you were young? (yes[a] or no)
		During the years you were growing up, had your female/male guardian often showed continued signs sadness or depression? (during all[a]. most[a]. some, or only a little of the childhood)
	Domestic violence	Have your father/mother ever beat up your mother/father? (often[a], sometimes[a], not very often, or never)
	Incarcerated household member	During the years you were growing up, have your female/male guardian ever been arrested or sent poison?(yes[a] or no)
	Parental separation divorce	Were your biological parents divorced (including long separation due to emotional problems) before you were 17 years? (yes[a] or no)
Expanded ACEs	Unsafe neighborhood	Was it safe being out alone night in the neighborhood where you lived as a child? (very safe,somewhat safe, not very safe[a], not safe at all[a])
	Bullying	When you were a child, how often were you picked on or bulled by kids in your neighborhood? (often[a] sometimes[a], not very often, or never)
		When you were a child, how often were you picked on or bullied by kids in your school? (often[a],sometimes[a],, not very often, or never)
New ACEs	Parental death	Either of the parents was dead before participant was 17 years. (yes[a] or no)
	Sibling death	Any of the siblings was dead before participant was 17 years. (yes[a] or no)
	Parental disability	Did your female/male guardian have a long time being sick on bed when you were young? (yes[a] or no)
		Did your female/male guardian have a serious deformity when you were young? (yes[a] or no)

a Answers indicate thresholds for ACEs.

Table S2 Association between ACEs and depressive-symptoms trajectory

ACEs	OR = e^ β (95% CI)[a]			
	Decreasing symptoms	High symptoms	Remitting symptoms	Increasing symptoms
0	1.00	1.00	1.00	1.00
1	1.18(0.03,0.30)	1.35(0.10,0.50)	0.98(-0.15,0.10)	1.08(-0.07,0.22)
2	1.45(0.23,0.51)	2.03(0.50,0.91)	1.16(0.01,0.28)	1.38(0.17,0.48)
3	1.82(0.43,0.76)	3.12(0.92,1.36)	1.40(0.17,0.49)	1.50(0.22,0.59)
≥4	2.52(0.75,1.10)	5.20(1.42,1.88)	1.87(0.45,0.80)	1.95(0.46,0.87)

[a]Reported 95% CI correspond to β ; significance threshold = 0.

Table S3 Association between 12 domains of ACEs and depressive-symptoms trajectory

Domains		OR = e^ β (95% CI)[a]			
		Decreasing symptoms	High symptoms	Remitting symptoms	Increasing symptoms
Physical abuse	no	1.00	1.00	1.00	1.00
	yes	1.01(-0.10,0.13)	0.98(-0.17,0.14)	0.93(-0.18,0.05)	0.97(-0.16,0.10)
Emotional neglect	no	1.00	1.00	1.00	1.00
	yes	0.96(-0.15,0.07)	1.00(-0.15,0.14)	1.04(-0.07,0.14)	0.99(-0135,0.11)
Household substance abuse	no	1.00	1.00	1.00	1.00
	yes	0.80(-0.42,-0.02)	0.75(-0.55,-0.02)	0.75(-0.50,-0.08)	0.79(-0.46,-0.01)
Household mental illness	no	1.00	1.00	1.00	1.00
	yes	1.86(0.47,0.77)	3.42(1.06,1.40)	1.81(0.45,0.75)	1.56(0.27,0.62)
Domestic violence	no	1.00	1.00	1.00	1.00
	yes	1.18(-0.02,0.36)	1.27(0.00,0.47)	0.94(-0.27,0.14)	1.21(-0.02,0.41)
Incarcerated household member	no	1.00	1.00	1.00	1.00
	yes	0.65(-1.51,0.65)	0.27(-3.33,0.72)	2.39(0.16,1.58)	1.63(0.38,1.35)
Parental separation divorce	no	1.00	1.00	1.00	1.00
	yes	1.68(0.07,0.97)	1.08(-0.58,0.74)	0.91(-0.66,0.47)	1.52(-0.10,0.94)
Unsafe neighborhood	no	1.00	1.00	1.00	1.00
	yes	1.15(-0.04,0.32)	1.46(0.17,0.59)	1.09(-0.09,0.27)	1.41(0.15,0.53)
Bullying	no	1.00	1.00	1.00	1.00
	yes	1.12(-0.03,0.26)	1.31(0.09,0.45)	1.13(0.02,0.27)	1.08(-0.09,0.24)
Parental death	no	1.00	1.00	1.00	1.00

	yes	1.38(0.20,0.44)	1.67(0.36,0.67)	1.19(0.05,0.30)	1.31(0.13,0.41)
Sibling death	no	1.00	1.00	1.00	1.00
	yes	0.90(-0.24,0.02)	0.80(-0.40,0.05)	0.88(-0.26,0.01)	0.85(-0.31,-0.01)
Parental disability	no	1.00	1.00	1.00	1.00
	yes	0.90(-0.32,0.11)	0.63(-0.77,-0.15)	0.89(-0.34,0.10)	0.77(-0.52,0.01)

[a]Reported 95% CI correspond to β; significance threshold = 0.
